# The Regulation of Nitrate Reductases in Response to Abiotic Stress in Arabidopsis

**DOI:** 10.3390/ijms23031202

**Published:** 2022-01-21

**Authors:** Xianli Tang, Yang Peng, Zheng Li, Hongwei Guo, Xinli Xia, Bosheng Li, Weilun Yin

**Affiliations:** 1 Beijing Advanced Innovation Center for Tree Breeding by Molecular Design, National Engineering Laboratory for Tree Breeding, College of Biological Sciences and Technology, Beijing Forestry University, Beijing 100083,China; txianli@163.com (X.T.); zhengli@bjfu.edu.cn (Z.L.); 2 Department of Biology, School of Life Sciences, Institute of Plant and Food Science, Southern University of Science and Technology, Shenzhen 518055, China; 11752009@mail.sustech.edu.cn (Y.P.); guohw@sustech.edu.cn (H.G.); 3 Key Laboratory of Molecular Design for Plant Cell Factory, Guangdong Higher Education Institute, School of Life Sciences, Southern University of Science and Technology, Shenzhen 518055, China

**Keywords:** NIA1, NIA2, ABA, protein degradation, NaCl

## Abstract

The two homologous genes, *NIA1* and *NIA2*, encode nitrate reductases in *Arabidopsis*, which govern the reduction of nitrate to nitrite. This step is the rate-limiting step of the nitrate assimilation and utilization. Therefore, the regulation of *NIA1* and *NIA2* is important for plant development and growth. Although they are similar in sequence and structure, their regulations are different. Genetic analysis uncovers that NIA1, rather than NIA2, plays a predominant role in adopting to ABA stress. Although both long-term stress conditions can cause an improvement in NIA1 levels, a decrease in NIA1 levels under short-term treatments seems to be necessary for plants to switch from the growth status into the adopting status. Interestingly, the downregulation of the NR is distinct under different stress conditions. Under ABA treatment, the NR proteins are degraded via a 26S-proteasome dependent manner, while the transcriptional regulation is the main manner to rapidly reduce the NIA1 levels under nitrogen deficiency and NaCl stress conditions. These results indicate that under stress conditions, the regulation of NIA1 is complex, and it plays a key role in regulating the balance between growth and adaptation.

## 1. Introduction

Nitrogen is a major restricting factor for plant growth. Most land plants obtain organic nitrogen and inorganic nitrogen from the soil [1]. Higher plants absorb two main sources of inorganic nitrogen, including nitrate and ammonium. Plants selectively absorb one of the inorganic nitrogen sources according to their species and the surrounding soil environment. In reduced and anaerobic soil conditions, the pH is relatively low and plants tend to absorb NH_4_^+^, while NO_3_^−^ is the main form of absorption in an aerobic soil environment with a higher pH [2,3,4]. 

The level of extracellular nitrate is important for normal plant growth because it provides the basic materials for normal biochemistry processes in cells [5,6]. The absorption of extracellular nitrate is through the nitrate transporter (NRT), and the nitrate is gradually reduced to nitrite by nitrate reductases (NRs) in the cytoplasm. Nitrite is further transported to the plastid by the nitrite transporter (NiRT2) and reduced to NH_4_^+^ by the nitrite reductases (NiR). The NH_4_^+^ can be used in the biosynthesis of amino acids with the help of the glutamine synthase (GS)/glutamine oxoglutarate amino transferase cycle (GOGAT). In a word, the transformation of inorganic nitrogen into organic nitrogen is essential for the de novo biosynthesis of various proteins in plants [7,8,9]. Among the complex assimilations and utilizations of nitrate, the rate-limiting step in the process is the reduction of nitrate to nitrite by nitrate reductases. In *Arabidopsis*, *NIA1* and *NIA2* are two homologous genes in charge of the reduction of nitrate to nitrite. Although they are similar in sequence, their nitrate reductase activity is different. NIA1 is responsible for 10% of the total nitrate reductase activity, while NIA2 takes up about 90% [10]. The null-allelic mutant *nia1-3nia2-1* is lethal, which can be rescued by the treatment of ammonium succinate, indicating that their functions of nitrite reductases are indispensable for normal plant growth and development (Figure S1). However, the single mutation of either *NIA1* or *NIA2* does not severely affect plant growth or development, indicating that the low level of nitrate reductase activity might be sufficient. A case in point is that the knock down mutant *nia1-1nia2-5,* which only harbors about 1% of nitrate reductase activity, can also complete the entire growth cycle [11]. Hence, the activity of the nitrate reductase is widely regulated in the plant to meet the requirement of normal plant growth. The primary one is the level of nitrate, which is the substrate of NRs. Although the supplementation of extra nitrate does not affect the expression level of NRs, the utilization of nitrate after long-term nitrogen deficiency can trigger the accumulation of NR transcripts [12]. 

In addition to the level of nitrate, many other factors can also affect the expression level or the activity of the nitrate reductases, including the light signaling [13], the circadian rhythms [14], the level of CO_2_ [15], the ABA signaling [16], the ethylene signaling [17], the cytokinin signaling [18] and nitrogen metabolites (mainly glutamine) functioning as negative regulators [5,9]. Over the past few decades, a variety of transcription factors have been identified in modulating the expression level of *nitrate reductase (NR)*. NODULE-INCEPTION-like proteins (NLPs) can sense the level of nitrate to induce the expression of *NIA1* and *NIA2*. The NLPs-mediating transcriptional network also includes many other nitrate-inducible genes, including the nitrate assimilation associated genes, the nitrate transporters and nitrite transporters [19,20]. The first identified gene of the *NLP* family is *NODULE INCEPTION* (*NIN*) in *Lotus japonicus*, which contained a conserved, plant unique RWP-RK DNA-binding domain [21]. The homologous genes of *NLP* are present in higher plants. There are nine *NLP* genes in the *Arabidopsis* genome, in which *NLP6* and *NLP7* are the two major players in primary nitrate responses [19,20,21]. Under nitrate treatment, NLP7 can translocate into the nuclear region, and binds to the nitrate response element (NRE) to activate the expression of nitrate response genes [22]. The nitrate nuclear retention of NLP6/7 is responsible for the transcriptional regulation of *NIA1* and *NIA2*, which plays an especially important role in plant growth and development. 

In addition, the post-transcriptional modulations in the activity of NR have also been reported, especially phosphorylation and the small ubiquitin-related modifier (SUMO) modification [23,24]. NR protein can be modulated by phosphorylation on the serine residues. The phosphorylated NR proteins are recognized by the 14-3-3 complexes, which can be further degraded by the 26S proteasomes [25]. According to the modification status, there are three main types of NR in plants, including the NR-free, NR-phosphorylation and NR-phosphorylation–14-3-3 complex. Among them, the NR-free usually have a higher enzyme activity, and the activity of the phosphorylated NR is strongly inhibited by the binding of the 14-3-3 complexes. In this manner, the activity and abundance of the NR are under strict control [26,27,28,29,30]. 

Interestingly, although *NIA1* and *NIA2* are highly similar, they are under very different regulations, indicating their special roles in the distinct signaling cascade. A case in point is the role of light signaling in the regulation of their expression. Constant light exposure can trigger the upregulation of *NIA2*, but not *NIA1* in a *HY5/HYH* dependent manner [31]. NIA1 specially plays a role in the biosynthesis of NO, while NIA2 does not have such an ability [32]. Hence, the differential regulation of *NIA1* and *NIA2* is important for plants in their adaptation to various environments. In this research, we explore whether the regulation of NIA1 and NIA2 protein in *Arabidopsis* is different under abiotic conditions for better adaptation. It is interesting to observe that the sensitivity of the NIA1 and NIA2 proteins is different in responding to the ABA signaling. Under short-term ABA treatment, the NIA1 protein is degraded while the NIA2 protein is unaffected. However, the mutant *nia1-3* rather than the *nia2-1* showed hypersensitivity to the ABA treatment, indicating that the ABA-mediated degradation of the NIA1 protein seems to be important for plant adaptation. Considering that NIA1 rather than NIA2 is involved in the NO biosynthesis, we believe that the downregulation of the NIA1 protein may be involve in the NO signaling to help the adaption of plants under ABA treatment. The ABA-mediated down regulation of the NIA1 protein is dependent on the 26S proteasome. Next, we tested whether this method was widely used for plants to adapt to various abiotic stresses. However, the degradation of NIA1 protein was not observed in other abiotic treatments. Under short-term high salt or null-nitrogen treatment, plants usually transcriptionally downregulate the level of *NIA1*. After long-term abiotic treatment, the levels of *NIA1* and *NIA2* are recovered to a normal level. Hence, our research indicated that NIA1 protein is involved in responding to the stress environment and maintaining the balance between plant growth and adaptation, while NIA2 protein is more about regulating plant growth and maintaining the nitrogen source required for normal plant growth. 

## 2. Result

### 2.1. Mutation of NIA1 Is Hypersensitive under ABA Treatment

**Figure 1:**
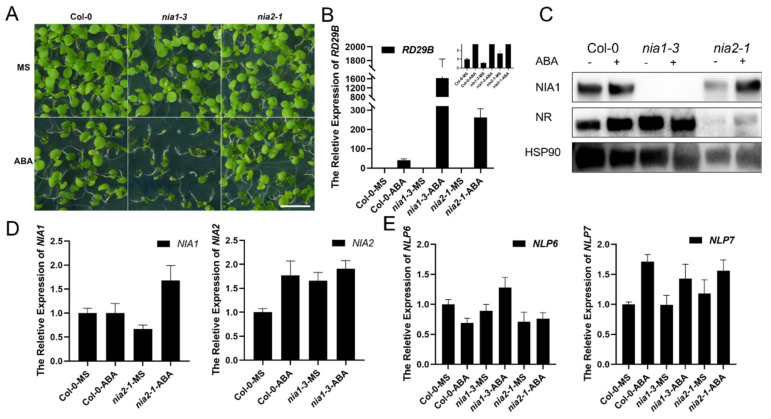
The *nia1-3* single mutant is hypersensitive to long-term ABA treatment. (**A**) Green seedlings that are 10 days old in the indicated background with or without ABA treatment. The scale bar is 0.5 cm; (**B**) gene expression analysis of *RD29b* transcripts in the indicated background with or without ABA treatment. Three biological repeats; (**C**) Western blot of the proteins extracted from samples in A by the NIA1 antibody and NR antibody. The quantification of relative levels of NIA1 or NR proteins are shown below; (**D**) gene expression analysis of *NIA1* or *NIA2* transcripts in the indicated background with or without ABA treatment. Three biological repeats; (**E**) gene expression analysis of *NLP6* or *NLP7* transcripts in the indicated background with or without ABA treatment. Three biological repeats.

It was reported that environmental signals, such as light exposure, can differentially regulate the expression of *NIA1* and *NIA2* for better adaptation [31]. Hence, we firstly asked whether the regulation of NR was also involved in the abiotic stress responses. ABA is a kind of well-known stress phytohormone, which can be induced by various abiotic environmental stresses, including drought and salt stress [33]. Seeds of Col-0, *nia1-3* and *nia2-1* were sown on the MS medium supplemented with or without 1 μM of ABA. Interestingly, after the long-term ABA treatment and incubation for 10 days, we found that the single mutant *nia1-3* showed a hypersensitivity to ABA while the seedlings of the single mutant *nia2-1* and Col-0 were indistinguishable (Figure 1A). The *RD29b* was a well-known responsive gene of ABA signaling, and its expression level was widely used to reflect the intensity of ABA signaling [34,35]. Under long-term ABA treatment, the expression level of *RD29b* was correspondingly elevated in Col-0 and the mutants. Among them, *nia1-3* displayed an extreme upregulation of *RD29b*, which was consistent with its hypersensitivity to ABA treatment (Figure 1B). Hence, the lethal phenotype of *nia1-3* might be a reason for the overactivated ABA signaling. The upregulation of *RD29b* in both *nia1-3* and *nia2-1* under ABA treatment, indicated that both NIA1 and NIA2 may play an inhibitory role in ABA signaling, while NIA1 was more essential. The results of Western blot assays indicated that, under long-term treatment, the accumulation of the NIA1 protein was negatively associated with the ABA sensitivity. In the wild-type and *nia2-1* mutants, they displayed an ABA tolerance phenotype and accumulated NIA1 proteins, while in the ABA hypersensitive *nia1-3*, the level of the NIA2 proteins was not affected (Figure 1C). 

These results indicate that the accumulation of NIA1 protein might be conducive for plants to fight against the long-term ABA treatment. The further exploration of the accumulation of NIA1 proteins allowed is to detect the expression levels of *NIA1*, *NIA2* and their transcriptional regulators *NLP6* and *NLP7*, with or without ABA treatment. Under ABA treatment, the expression level of *NIA1*, rather than *NIA2*, was elevated in both the wild-type and *nia2-1* (Figure 1D). To further study the role of transcriptional regulation in NIA1 in responding to ABA treatment, we detected the expression levels of *NIA1* and *NIA2* in the *nlp6* or *nlp7* single mutant under long-term ABA treatment. The results showed that neither the *nlp6* nor *nlp7* mutant showed hypersensitive to ABA treatment (Figure S2A). The Western blot assays further indicated that although the NIA1 protein level was low in *nlp7*, it still displayed a protein accumulation pattern under long-term ABA treatment (Figure S2B). In both *nlp6* and *nlp7*, the long-term ABA treatment can still promote the increase in the transcription of NIA1, which can contribute to the accumulation of the NIA1 protein (Figure 1E). These results indicate an association between the accumulation of NIA1 protein and the ABA sensitivity of plants. It seems that the dynamic regulation of the NIA1 protein was essential for the adaption of plants in responding to ABA treatment. In *nlp6* and *nlp7*, it also can accumulate more NIA1 under long-term ABA treatment. Under long-term ABA treatment, plants finally accumulated the NIA1 protein via a transcriptional network that was limited. Hence, we asked whether if it was the dynamic regulation of the NIA1 protein level rather than the accumulation of NIA1 protein that played an essential role in plant adaptation. 

### 2.2. ABA Treatment Rapidly Regulates the Abundance of NR Proteins via a 26S Proteasome Manner

**Figure 2:**
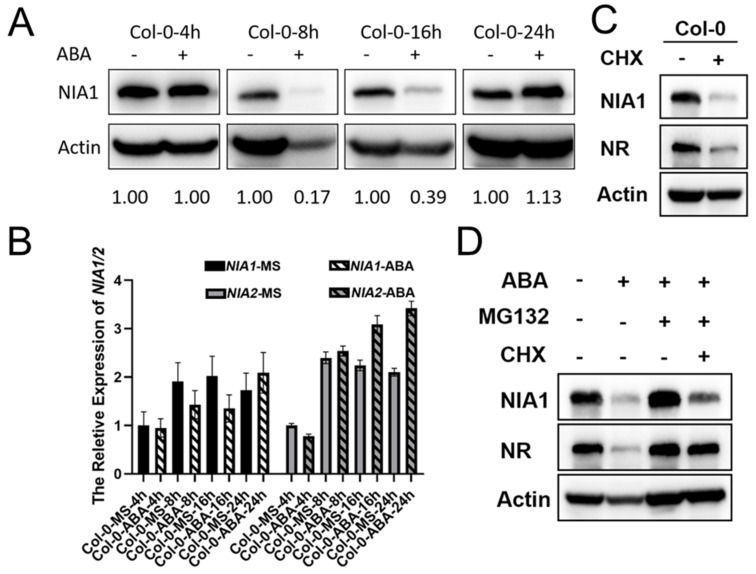
NR proteins are degraded via a 26S proteasome under short-term ABA treatment. (**A**) Western blot of the proteins extracted from seedlings under indicated treatments by the NIA1 antibody and NR antibody. The quantification of relative levels of NIA1 proteins are shown below; (**B**) Western blot of the proteins extracted from seedlings under the indicated treatments by the NIA1 antibody and NR antibody. The quantification of relative levels of NIA1 proteins are shown below; (**C**) gene expression analysis of *NIA1* or *NIA2* transcripts in the indicated background with or without ABA treatment. Three biological repeats; (**D**) Western blot of the proteins extracted from seedlings under the indicated treatments by the NIA1 antibody and NR antibody. The quantification of the relative levels of NIA1 proteins are shown below.

To further dissect the regulatory network of ABA in NR proteins, we further detected the abundance of NIA1 protein under short-term ABA treatment. Green seedlings that were 8 days old in an MS medium were harvested and transferred into a liquid MS medium supplemented with 100 μM ABA or coincident DMSO. After the indicated treatment periods, the seedlings were harvested for further Western blot assays. It was interesting to observe that the NIA1 protein level greatly decreased after 8 h of ABA treatment, and it gradually recovered to a higher level under 24 h of ABA treatment (Figure 2A). Further qPCR assays uncovered that 8 h or 16 h of ABA treatment slightly decreased or did not affect the transcriptional levels of *NIA1* or *NIA2*, respectively, while the 24 h ABA treatment can elevate the expression levels of *NIA1* and *NIA2* (Figure 2B). The dominant absence of the NIA1 protein under 8 h or 16 h of ABA treatment cannot be fully explained by the slight downregulation of *NIA1* transcripts. Previous reports have uncovered that NIA1 usually undergoes a ubiquitination pathway to regulate its abundance. Consistently with this, we found that the new biosynthesis of NR proteins was blocked under CHX treatment, and both the NIA1 and NIA2 proteins were unstable (Figure 2C). Hence, we further tested whether short-term ABA treatment can rapidly induce the degradation of NR proteins to decrease their abundance. MG132 was a chemical that was widely used to inhibit the activity of the 26S proteasome. Under the combination of ABA and MG132 treatment, the downregulation of the NR protein that underwent 8 h of ABA treatment was rescued, indicating that the ABA-mediated downregulation of NR proteins might occur in a 26S proteasome-dependent manner (Figure 2D). These results indicated that under the short-term ABA treatment, plants can rapidly reduce the abundance of growth-promoting genes, including NR proteins. With the extension of ABA treatment, the growth-promoting genes were elevated transcriptionally to help the plants’ adaptation. Although both the NIA1 and NIA2 proteins were degraded under short-term ABA treatment, our genetic analysis indicated that the NIA1 proteins played a unique role in responding to the ABA treatment (Figure 1A). However, the underlying mechanisms are mainly unclear.

### 2.3. Single Mutation of NIA1 or NIA2 Shows a Similar Phenotype under Long-Term NaCl Stress

The differential sensitivity to ABA between the single mutant *nia1-3* and *nia2-1* further prompted us to ask whether they played a differential regulatory role in other kinds of abiotic stresses. The seeds of Col-0, the single mutant *nia1-3* and *nia2-1* were sown on, and then the MS medium with the gradient concentrations of NaCl were added. After incubation in the growth room for 10 days, the seedlings were imaged and harvested for further assays. 

**Figure 3:**
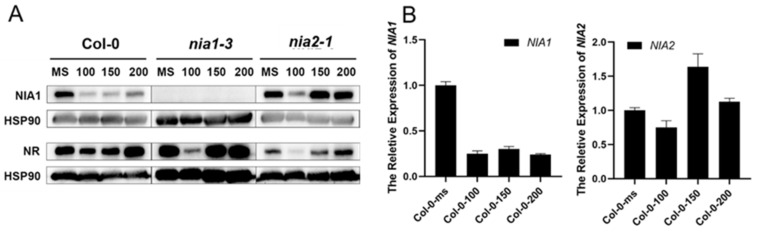
NaCl stress transcriptionally regulates the expression of NIA1 rather than NIA2. (**A**) Western blot of the proteins extracted from seedlings under the indicated treatments by the NIA1 antibody and NR antibody. The quantification of the relative levels of NIA1 or NR proteins are shown below; (**B**) gene expression analysis of *NIA1* or *NIA2* transcripts in Col-0 under indicated treatments. Three biological repeats.

Under 100 mM of NaCl or 150 mM of NaCl treatment, the seedlings of all the genotypes produced true leaves without obvious growth defects in *nia1-3* or *nia2-1*. When the concentration of NaCl was raised to 200 mM, the seedlings of all genotypes showed strong growth inhibition (Figure S3A). In Col-0, the expression level of *NIA1* clearly decreased under the NaCl treatment, while there were no significant changes in the expression level of *NIA2* (Figure 3B). Consistent with the results of qPCR, the NIA1 protein was also very low while the NIA2 protein was unaffected under NaCl treatment (Figure 3A). This result indicates that NaCl stress can strongly regulate the transcriptional level of NIA1 but not NIA2. In a word, long-term NaCl treatment can strongly downregulate the abundance of *NIA1* transcriptionally. 

**Figure 4:**
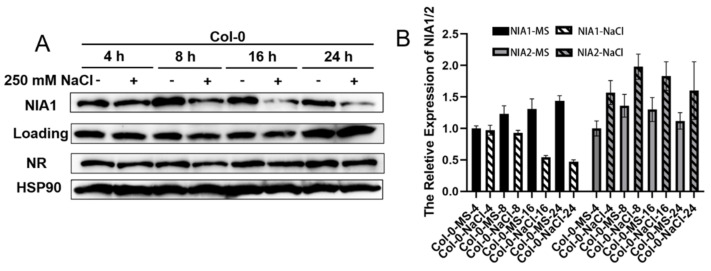
Short-term NaCl treatment transcriptionally downregulates NIA1 proteins rather than NIA2 proteins. (**A**) Western blot of the proteins extracted from seedlings under indicated treatments by the NIA1 antibody and NRs antibody. The quantification of the relative levels of NIA1 or NR proteins are shown below; (**B**) gene expression analysis of *NIA1* or *NIA2* transcripts in Col-0 under the indicated treatments. Three biological repeats.

To explore the transient response under NaCl treatment, 8-day-old green seedlings of Col-0, *nia1-3* and *nia2-1* were harvested and transferred into liquid MS medium supplemented with 250 mM of NaCl for time-course treatments. Under NaCl treatment, the results can be observed in the protein level of NIA1, rather than in the NIA2 protein, which had levels that were strongly downregulated under NaCl treatment for more than 8 h (Figure 4A). The corresponding results in the mRNA level showed that the expression level of *NIA1* was gradually downregulated, while the NIA2 transcript was elevated. These results together indicate that either short-term or long-term NaCl treatment can especially regulate the abundance of *NIA1* in the transcriptional levels. 

### 2.4. Nitrogen Deficiency Stress Also Dynamically Downregulates Both NIA1 and NIA2 in the Transcriptional Level

**Figure 5:**
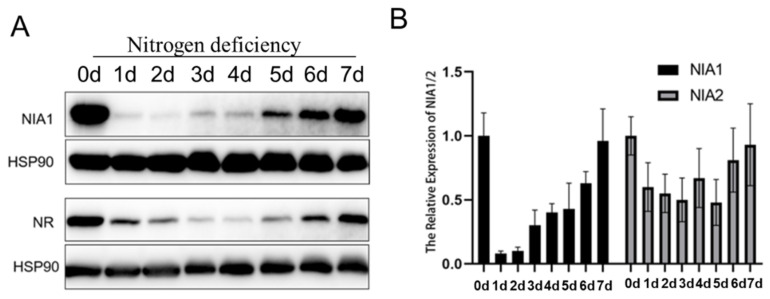
Nitrogen deficiency dynamically regulates the expression level of NIA1. (**A**) Western blot of the proteins extracted from seedlings under the indicated treatments by the NIA1 antibody and NR antibody. The quantification of relative levels of NIA1 or NR proteins are shown below; (**B**) gene expression analysis of *NIA1* or *NIA2* transcripts in Col-0 under the indicated treatments. Three biological repeats.

Nitrogen deficiency stress is also a common natural stress, and previous researches have uncovered that the expressions of *NIA1* and *NIA2* were immediately induced under the supplement of nitrate (Figure S4) [36], to detail the analysis of the expression of *NIA1* and *NIA2* under nitrogen deficiency treatment. The 5-day-old Col-0 seedlings in an MS medium were harvested and transferred to a nitrogen-deficient medium for 1 day to 7 days. After one-day nitrogen deficiency treatment, the expression level of *NIA1* rapidly decreased and then recovered slowly (Figure 5A). Consistent with the protein level, the *NIA1* mRNA disappeared firstly and gradually accumulated under long-term nitrogen deficiency (Figure 5B). However, the expression level of *NIA2* was downregulated and maintained at about 50% of the level under nitrogen deficiency. The expression of *NIA2* was rapidly induced by nitrate at a very low concentration (10^−6^ mM KNO_3_), but not in *NIA1* (Figure S4B). Hence, *NIA2* was more efficient than *NIA1* in responding to the nitrate, while under the nitrogen deficiency condition the expression of *NIA1* was more sensitive than *NIA2*. Hence, the dynamic regulation of the expression of NIA1 is important for plants for their adaptation to nitrogen deficiency.

## 3. Discussion

**Figure 6:**
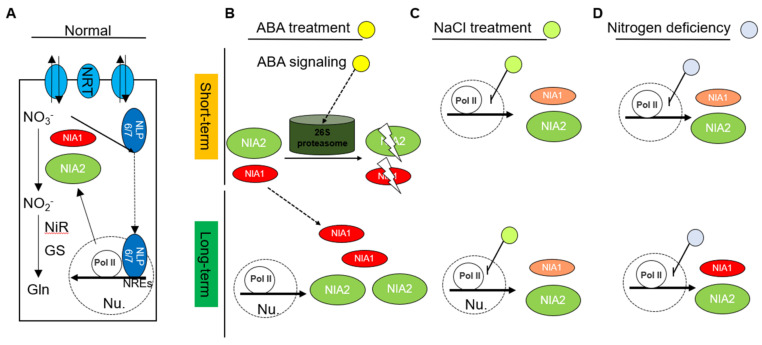
Schematic diagrams display the regulation of nitrate reductases in response to abiotic stress. (**A**) Under normal conditions, the expression levels of *NIA1* and *NIA2* are highly controlled in a transcriptional manner. Nitrate supplementation triggers the trans-localization and retention of NLP6/7 in the nuclear, in which they can bind to the NREs to promote the expression of *NIA1* and *NIA2*; (**B**) under short-term ABA treatment, the NIA1 protein and NIA2 protein are degraded via a 26S proteasome-dependent manner. Under long-term ABA treatment, the expression level of *NIA2* is upregulated, contributing to the accumulated NIA2 protein. Meanwhile, the long-term ABA treatment might gradually reduce the protein degradation of the NIA1 protein to promote its accumulation for better adaptation; (**C**) under short-term NaCl treatment, the expression level of *NIA1* is decreased, contributing to the disappearance of the NIA1 protein. The NIA1 protein will also gradually decrease with the extension of the treatment time, and *NIA2* has no obvious change. Under long-term salt stress, the decrease in the transcription of NIA1 and the disappearance of NIA1 proteins also cannot be recovered. In contrast, both the expression level and protein level of NIA2 are only partially reduced under short-term or long-term NaCl treatment; (**D**) under nitrogen deficiency conditions, both the expression and protein levels of NIA1 and NIA2 are strongly inhibited. Along with prolonging the nitrogen deficiency conditions, the expression and protein levels of NIA1 and NIA2 gradually recovered to the pre-treated levels.

The sessile plant usually encounters complex environments, including the biotic stresses and abiotic stresses. The abiotic stresses usually result from the adverse surrounding environmental conditions, including the lack of nutrients and the accumulation of stressful substances. Plants usually overcome the adversity by activating the ABA-mediated signaling pathway. Therefore, ABA can be considered as a kind of stress phytohormone [37]. In their adaptation to adversity, plants usually utilize a two-step strategy, in which the plant firstly inhibits the growth stage and gradually recovers after they are become comfortable. This two-step strategy is certified by a collaboration of complex regulatory networks, including the transcriptional reprogram, the transitional reprogram and protein aggregation or degradation [38]. As the rate-limiting enzymes in nitrate assimilations, the *NIA1* and *NIA2* are essential growth-related genes for plant development. In this work, we systematically analyzed the regulation of nitrate reductases in responding to short-term or long-term abiotic stresses (Figure 6). 

In normal conditions, the expression levels of *NIA1* and *NIA2* are highly controlled in a transcriptional manner. Nitrate supplementation triggers the retention of NLP6/7 in the nuclear region, in which they can bind to the NREs to promote the expression of *NIA1* and *NIA2* [19]. Consistently with this, we also found that under nitrogen deficiency conditions, both the expressions of the mRNA level and protein level of NIA1 and NIA2 are strongly inhibited. Along with prolonging the nitrogen deficiency conditions, the mRNA and protein levels of NIA1 and NIA2 gradually recovered to their pre-treated levels, indicating that plants gradually release the transcriptional inhibition on NR after adaption. 

Interestingly, the distinct regulations between *NIA1* and *NIA2* exist under other kinds of stresses. Under short-term ABA treatment, although the expression levels of NIA1 and NIA2 do not show a strong inhibition, the NIA1 proteins and NIA2 proteins disappear. Combining the treatment of ABA and MG132 can restore the disappearance of NIA1 proteins and NIA2 proteins, indicating that ABA can mediate the degradation of NIA1 protein and NIA2 protein via a 26S proteasome-dependent manner. Under long-term ABA treatment, the mRNA expression level of *NIA2* is upregulated, contributing to the accumulated NIA2 protein. However, under long-term ABA treatment, the accumulation of the NIA1 protein is not due to the upregulation of *NIA1* in the transcriptional level. Hence, the long-term ABA treatment might gradually reduce the protein degradation of the NIA1 protein to promote its accumulation for better adaptation. The single mutation of *NIA1* shows a hypersensitivity to long-term ABA treatment, implying a unique role of the NIA1 protein in ABA responses. Our observations uncovered a complex regulatory role of ABA in nitrate reductases. Mutually, the NIA1 seems to play a role in ABA signaling. A reasonable possibility is that NIA1 also acts in the biosynthesis of nitric oxide (NO) [39]. A point in case is that the loss of nitrate reductases might alter the ABA signaling, resulting in the stomatal closure defect [16]. This evidence will yield more attention in this area. 

We also explored the regulation of nitrate reductases under short-term and long-term NaCl treatment. Under short-term NaCl treatment, the expression level of *NIA1* decreased, contributing to the disappearance of the NIA1 protein. The NIA1 protein will also gradually decrease with the extension of the treatment time, and NIA2 has no obvious change. Under long-term salt stress, the decrease in the transcription of *NIA1* and the disappearance of the NIA1 proteins cannot be recovered. In contrast, both the expression level and protein level of NIA2 are only partially reduced under short-term or long-term NaCl treatment. 

Taken together, our researches indicate that the regulation of NIA1 and NIA2 is complex and distinct under different kinds of abiotic stresses. Generally, the ABA treatment can regulate the protein abundance of NIA1 and NIA2 to help plants adapting to stressful environments. Salt stress and nitrogen deficiency treatment mainly downregulate the transcriptional levels to help plant adaption. After long-term stress treatment, the mRNA expression level or the protein level of NR are usually reset to similar levels, compared to the pre-treat conditions, indicating that plants can fine-tune the efficiency of nitrate assimilation for better adaptation. In addition, it is interesting to find that the regulation of *NIA1* and *NIA2* is different under abiotic stress. Both the transcriptional regulation and post-transcriptional regulation of NIA1 are more sensitive than NIA2 under abiotic stresses. However, either the amount or the ability of the NIA1 nitrate reductase are much lower than NIA2. It is worth further exploring the role of NIA1 in signaling or other biochemistry pathways under abiotic stresses. 

## 4. Materials and Methods

### 4.1. Plant Material

*Arabidopsis* plants were all Columbia ecotypes. Samples of *nia1-3* (Salk_148487C) *nia2-1* (Salk_138297C), *nlp6* (Salk_018362C) and *nlp7* (Salk_026134C) were obtained from the Arabidopsis Biological Resource Center (https://abrc.osu.edu/, accessed on 30 December 2021). 

### 4.2. Growth Condition

For short-term ABA treatment, the seedlings were cultivated on the MS medium for 8 days, and seedlings were transferred into liquid MS medium supplemented with 100 μM of ABA or the equal volume of DMSO in the 6-well cell, respectively. 

For short-term NaCl treatment, the seedlings were grown on the MS medium for 8 days, and seedlings were transferred into liquid MS medium supplemented with 250 mM of NaCl. To avoid dehydration, the roots were placed on the gauze and immersed in the salt water while the ground part was far away from the NaCl medium.

For long-term treatment, seeds of different genotypes were sowed on the growth medium supplemented with 1 μM of ABA that was different for 10 days or with different concentrations of NaCl for 10 days. Then, the seedlings were harvested for further protein or RNA extraction.

For the nitrogen deficiency experiment, the seeds were germinated and grown on the MS medium for 5 days. Then, the seedlings were transferred to the nitrogen-deficient medium for one to seven days. The nitrogen deficient medium was adopted from the nitrogen-depleted MS basal salt from Phyto Technology (catalogue numbers M531).

### 4.3. Gene Expression Analysis by Quantitative PCR

The methods of total RNA extraction, reverse transcription and real-time PCR were previously described [40]. All the genotype identification primers and gene-specific primers used are listed in the Supplementary Table S1. 

### 4.4. Total Protein Extraction and Immunoblot Assays

For the detailed protein extraction and immunoblot assays, please refer to the published article [40]. 

## Supplementary Materials

The following supporting information can be downloaded at: https://www.mdpi.com/article/10.3390/ijms23031202/s1.
