# Supplementary material for: The Regulation of Nitrate Reductases in Response to Abiotic Stress in Arabidopsis
Source: Int J Mol Sci. 2022 Jan 21;23(3):1202. doi: 10.3390/ijms23031202 (PMC8835330; doi:10.3390/ijms23031202)
Supplement: Supplementary file 1 [file ijms-23-01202-s001.zip › ijms-1560708-supplementary.pdf]

Supplement Figure

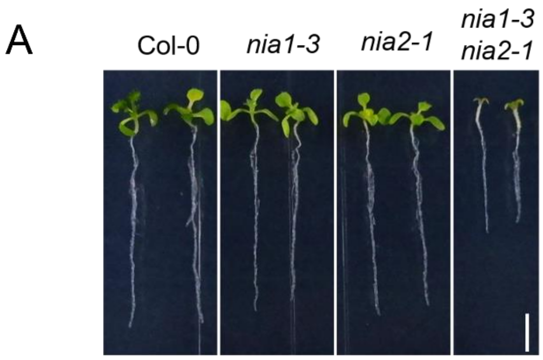

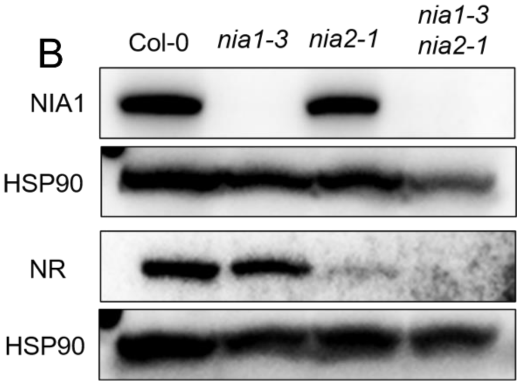

Figure S1. The null-allele mutant *nia1nia2* was lethal.

A. The phenotype of the seedlings in indicated background. The scale bar is 0.5cm.

B. Western blot of the proteins extracted from samples in **A** by NIA1 antibody and NR antibody to test their specialty.

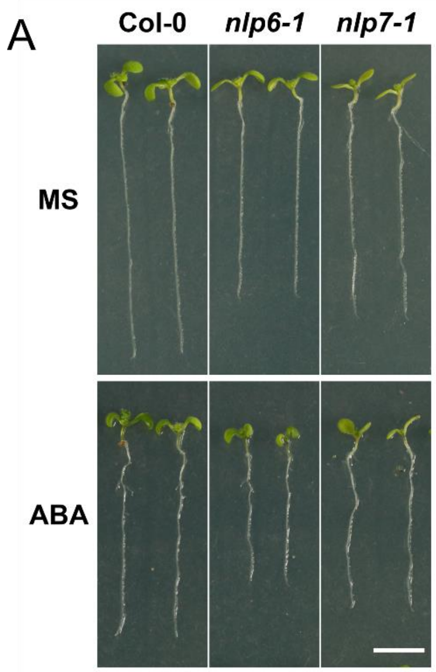

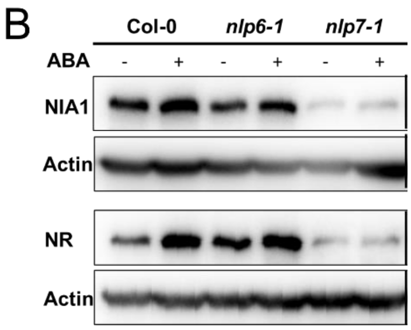

Figure S2. Transcription regulation was less important in response to ABA.

A. The phenotype of the 10-day-old green seedlings in indicated background with or without ABA treatments. The scale bar is 0.5 cm.

B. Western blot of the proteins extracted from samples in **A** by NIA1 antibody and NR antibody.

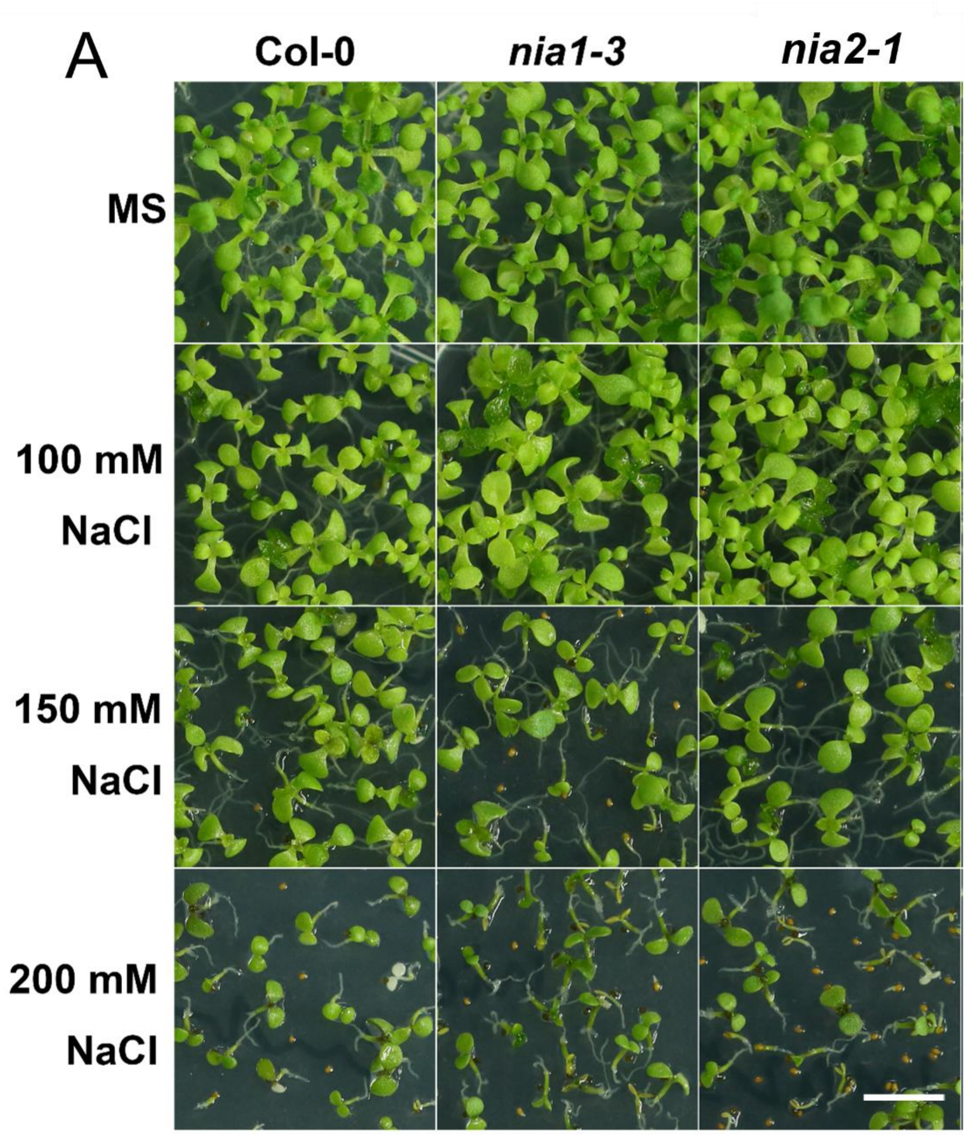

Figure S3. Long-term NaCl treatment inhibits plant growth in Col-0, *nia1-3,* and *nia2-1*.

The phenotype of the 13-day-old green seedlings in indicated background grown in MS medium supplementing with gradient concentrations of NaCl. The scale bar is 0.5 cm.

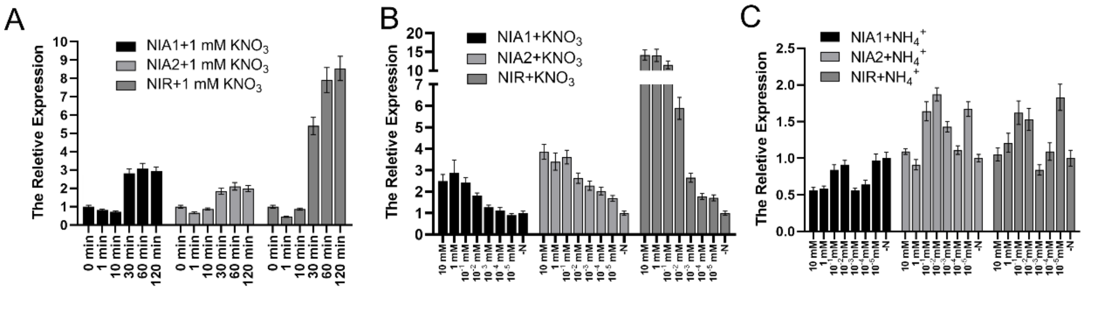

Figure S4. *NIA1* and *NIA2* can be induced by nitrate timely and efficiently.

A-C. Gene expression analysis of *NIA1* and *NIA2* transcripts in Col-0 under indicated treatments. Three biological repeats.
